# The Associations of Sensory Impairment with 10‐Year Risk of Dementia and Alzheimer’s Disease: The Health and Retirement Study, 2010‐2020

**DOI:** 10.1002/alz.088545

**Published:** 2025-01-09

**Authors:** Kun Li, Rahul Ghosal, Donglan Zhang, Yike Li, Matthew Lohman, Monique J Brown, Anwar T Merchant, Chih‐Hsiang Yang, Jean Neils‐Strunjas, Daniela B. Friedman, Jingkai Wei

**Affiliations:** ^1^ Duke University, Washington, DC USA; ^2^ University of South Carolina, Columbia, SC USA; ^3^ New York University Long Island School of Medicine, Mineola, NY USA; ^4^ Vanderbilt University Medical Center, Nashville, TN USA

## Abstract

**Background:**

Although visual and hearing impairment have been identified as established risk factors for dementia, evidence was limited on the association of the coexistence of these two sensory impairments with incident dementia, especially in the U.S. The study aimed to examine the associations between sensory impairment and 10‐year risk of dementia or Alzheimer’s disease.

**Method:**

A prospective cohort study was designed based on the Health and Retirement Study from 2010 to 2020. Individuals aged 50 years and older without self‐reported dementia and Alzheimer’s disease were included in the analysis. Information of self‐reported visual and hearing impairment was obtained at Wave 10 in 2010, and participants were categorized as normal, with visual impairment only, hearing impairment only, and dual sensory impairment. Main failure events included self‐reported incident dementia and Alzheimer’s disease over a 10‐year follow‐up period. Fine‐Gray competing risk regression models were applied for the associations of sensory impairment with incident dementia and Alzheimer’s disease, adjusted for demographic characteristics, health behaviors, and health conditions at baseline.

**Result:**

Of 20,248 identified individuals, 14.6% had visual impairment only, 11.2% had hearing impairment only, and 9.1% had dual impairment at baseline. After adjustment for all covariates, dual sensory impairment was associated with higher risk of dementia (hazards ratio (HR) = 1.46, 95% CI: 1.23‐1.73) and Alzheimer’s disease (HR = 1.35, 95% CI: 1.03‐1.76). Visual impairment only was associated with incident dementia (HR = 1.98, 95% CI: 1.38‐2.83) and Alzheimer’s disease (HR = 2.99, 95% CI: 1.60‐5.62) among individuals <65 years. No association was observed between hearing impairment only and incident dementia or Alzheimer’s disease.

**Conclusion:**

Older adults in the U.S. with visual and hearing impairments simultaneously had a particularly greater risk of dementia and Alzheimer’s disease, and having visual impairment alone was associated with an elevated risk of dementia; the associations were stronger among people <65 years.

